# Scleral changes in systemic lupus erythematosus patients using swept source optical coherence tomography

**DOI:** 10.3389/fimmu.2023.1278893

**Published:** 2023-11-02

**Authors:** Lulu Chen, Lihui Meng, Lu Sun, Youxin Chen

**Affiliations:** ^1^ Department of Ophthalmology, Peking Union Medical College Hospital, Chinese Academy of Medical Sciences, Beijing, China; ^2^ Key Laboratory of Ocular Fundus Diseases, Chinese Academy of Medical Sciences and Peking Union Medical College, Beijing, China

**Keywords:** scleral thickness, systemic lupus erythematosus, SS-OCT, preclinical change, disease duration

## Abstract

**Purpose:**

This study aims to examine scleral thickness in patients with systemic lupus erythematosus (SLE) without clinically evident scleritis and episcleritis, utilizing swept-source optical coherence tomography (SS-OCT).

**Methods:**

This cross-sectional single center study compared scleral thickness (Nasal scleral thickness 1mm, 2mm, 3mm, 6mm from scleral spur; Temporal scleral thickness 1mm, 2mm, 3mm, 6mm from scleral spur) in 73 SLE patients without clinically evident scleritis and episcleritis and 48 healthy volunteers with SS-OCT. Further, we investigated the correlation between scleral thickness in SLE patients and various parameters including laboratory markers, disease duration, disease activity, and organ involvement.

**Results:**

Across all measured sites (nasal scleral thickness at distances of 1mm, 2mm, 3mm, and 6mm from the scleral spur, and temporal scleral thickness at the same distances), the scleral thickness in the SLE group was significantly greater than that in the control group (all p-values <0.001). SLE patients with a disease duration of 5 years or less exhibited a higher scleral thickness compared to those with a more prolonged disease duration. Patients with a higher erythrocyte sedimentation rate (ESR) had a thinner temporal scleral thickness. However, no significant associations were identified between scleral thickness and disease activity, organ involvement, or other laboratory markers.

**Conclusion:**

Scleral thickness measured by SS-OCT was higher in SLE patients than healthy controls. Changes in scleral thickness in SLE patients are related to disease duration and ESR. SS-OCT can detect asymptomatic structural changes in SLE patients and may be a useful tool in the evaluation of early scleral abnormality.

## Background

Systemic lupus erythematosus (SLE) is a complex autoimmune disease that involves multiple organs with remarkably heterogeneous clinical features. The etiology of SLE is still undefined, but various factors including genetic, epigenetic, immunoregulatory, environment, and infectious factors contribute to the onset of the disease (1). The prevalence of SLE in the population is 20-150 cases per 100 000 (2, 3), and about 3%~31% of patients may present with ocular involvement (4–9).

Occurrences of scleritis and episcleritis in SLE patients are relatively infrequent. The sclera is a resilient and structurally complex connective tissue that forms the outer layer of the eye. It consists of a scaffold of fibrous collagen in a hydrated interfibrillar matrix of proteoglycans and glycoproteins (10). Prior research has indicated that scleral layers may thicken in cases of scleral inflammation, as discerned through anterior segment optical coherence tomography (AS OCT) (11–13). These studies evidenced the potential for scleral tissue changes under local or systemic inflammatory conditions, but relationships between such changes and systemic conditions or laboratory factors remained unexplored. Subsequent studies revealed preclinical structural and microcirculatory alterations of the retina and optic nerve in SLE patients, indicative of structural impairment even in asymptomatic cases (14–17). A more recent study reported subclinical changes would also happen in scleral tissue in SLE patients with AS OCT (18). Comprehending the preclinical changes of the sclera and their associations with laboratory markers and systemic conditions may play a pivotal role in early detection and management of scleritis or other sclera-related conditions in SLE patients.

Swept-source OCT (SS-OCT) and spectral-domain OCT (SD-OCT) both employ Fourier-domain technology, but SS-OCT operates at longer wavelengths (1040-1060nm) than SD-OCT (840nm). SS-OCT offers several advantages over SD-OCT, including faster imaging speed, higher resolution, enhanced tissue penetration, and reduced imaging artifacts (19). It excels in demarcating the boundaries of anterior segment structures compared to SD-OCT (20). A few recent studies successfully measured scleral thickness in different study groups with the help of SS-OCT (21–23). However, to our knowledge, no study has yet assessed scleral thickness in SLE patients utilizing SS-OCT.

In our current study, we aimed to assess scleral thickness in SLE patients without clinically apparent scleritis and episcleritis via SS-OCT, and to examine its association with SLE laboratory markers, disease duration, disease activity, and organ involvement.

## Method

### Study population

This cross-sectional, observational study was conducted at a single center, the Department of Ophthalmology at Peking Union Medical College Hospital (PUMCH), from December 1, 2022, to May 24, 2023. It involved 74 SLE patients who were consecutively recruited at outpatient clinics or during inpatient ophthalmic consultations. These patients were diagnosed according to the 2019 ACR/EULAR classification criteria for SLE. Exclusion criteria included age below 18 years, spherical equivalent exceeding ±4.00 diopters, history of ophthalmic diseases (such as glaucoma, uveitis, choroidal neovascularization, central serous choroidopathy, and trauma), history of ocular surgery (including cataract surgery, scleral buckling, and vitrectomy), poor OCT image quality, coexistence of other autoimmune diseases, and presence of active scleritis or episcleritis at examination. For patients highly suspicious of posterior scleritis, ultrasonography was performed to confirm the diagnosis. Once the patient was diagnosed with posterior scleritis, the patient was excluded from the study. The control group consisted of 48 healthy volunteers who attended regular ophthalmic examinations at PUMCH, matched for age, gender, and spherical equivalent with the SLE group. Subjects in the control group have no previous ocular diseases which could affect scleral measurements. The study adhered to the tenets of the Declaration of Helsinki and received approval from the Institutional Board of Peking Union Medical College Hospital(I-22PJ1024). All participants provided informed consent.

Clinical manifestations at diagnosis, disease duration, and current medication usage were noted for each SLE patient. Laboratory markers, including complete blood count (CBC), erythrocyte sedimentation rate (ESR), high-sensitivity C-reactive protein (hs-CRP), creatinine, antinuclear antibody (ANA), anti-dsDNA, complement fractions (C3 and C4), and aPLs (including LA, IgG and IgM isotypes of aCL and anti-beta-2-glycoprotein I (ab2GPI) antibodies) were recorded. SLE activity was quantified using the SLE Disease Activity Index-2000 (SLEDAI-2K), and the results were classified as follows (1): 0-6 indicated inactivity or mild activity; (2) 7-12 suggested moderate activity; (3) ≥13 represented severe activity.

All enrolled participants underwent comprehensive ophthalmic evaluations, including best corrective visual acuity (BCVA), intraocular pressure (IOP), slit-lamp examination of the anterior segment and fundus, and swept-source OCT (BM-400K BMizar; TowardPi Medical Technology, Beijing, China) of the anterior segment. One eye from each participant was randomly selected for further analysis.

### SS-OCT image acquisition

The anterior segment OCT (AS OCT) system was used to capture the image of sclera. The system applies the wavelength centered at 1060 nm, and the scan rate is 40 000 A-scans per second. The axial resolution is 3.8μm and the lateral resolution is 10μm. The patient was positioned in front of the device with chin and forehead firmly fixed to the device to minimize head or eye position changes during the examination. The participant was first asked to gaze the internal fixation target and a single 16.5mm line scan centered at the cornea was performed. Then the participant was asked to adopt a maximal temporal or nasal gaze at an external fixation target during scanning of the temporal or nasal sclera. A raster scan with scan field of 16mm×12mm scanning parallel to the rectus muscle was performed to get the image of the temporal or nasal sclera. After reviewing all the scanned images, we selected one image with best quality of the horizontal section perpendicular to the corneal limbus for further analysis.

### SS-OCT measurements

All cases were measured under optical coherence tomography standard rules and policies. The cornea thickness was defined as the distance between the two hyper reflective band demarcating the boundaries of the cornea. The anterior chamber depth was defined as the distance between the internal limit of the cornea to the surface of the lens. Cornea thickness and anterior chamber depth were measured automatically by the built-in software.

**Figure 1:**
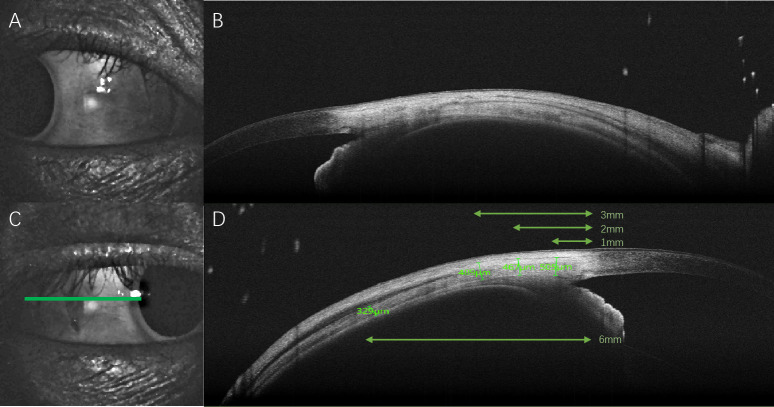
Illustration of the SS-OCT imaging for each participant. **(A)** Nasal scleral image was captured. **(B)** SS-OCT image of the nasal sclera. **(C)** Temporal scleral image was captured and the selected image with best quality of the horizontal section perpendicular to the corneal limbus (green line). **(D)** SS-OCT image of the temporal sclera with scleral thickness measured vertically at points 1mm (ST1), 2mm (ST2), 3mm (ST3), and 6mm (ST6) posterior to the scleral spur.

The scleral thickness was measured manually with the built-in caliber tool of the software. The external limit of the sclera was identified by the deep episcleral vascular plexus, which manifests as a thin hyporeflective region below the conjunctiva- Tenon capsule. The internal limit of the sclera presented as a sharply demarcated line between the hyper-reflective scleral tissue and the hyporeflective ciliary body tissue. We measured the temporal and nasal scleral thickness vertically at points 1mm (ST1), 2mm (ST2), 3mm (ST3), and 6mm (ST6) posterior to the scleral spur (**Figure 1**). Two independent examiners performed the measurement of scleral thickness and we used the average measurements for statistical analysis.

### Statistical analysis

Statistical analysis was performed using the software SPSS 25.0 (IBM, Chicago, United States), and *P <*0.05 was considered statistically significant. The normality of data was examined by the Shapiro–Wilk test. Continuous variables were presented as mean ± standard deviation. Analysis of variance (ANOVA) was applied to compare continuous variables among groups. Mann-Whitney U test was used to compare continuous variables between groups. The categorized data were evaluated with the chi-square test or Fisher exact test.

## Results

The study included 73 eyes from 73 SLE patients and 48 eyes from 48 healthy participants. 64 patients were female (87.7%), and the mean age was 36.5 ± 11.2 years. The mean SLE duration was 7.2 ± 5.9 years, and 60.3% (n=44) of patient had a disease duration more than 5 years. The average SLEDAI-2K score was 7.3 ± 7.6. The number of patients in the inactivity or mild activity group, moderate active group, and severe active group were 36 (49.3%), 20 (27.4%), and 17(23.3%). The main clinical manifestations were shown in **Table 1**.

**Table 1:** Demographic features, clinical manifestations, laboratory data, and treatment medication in SLE patients.

	SLE group (n=73)
Age, years (mean ± SD)	36.5 ± 11.2
Female sex, n (%)	64 (87.7)
Disease duration, years (mean ± SD)	7.2 ± 5.9
More than 5 years (%)	44 (60.3)
Less than 5 years, n (%)	29 (39.7)
SLE disease activity score(SLEDAI-2K) (mean ± SD)	7.3 ± 7.6
SLEDAI score grouping (mild/moderate/severe)	36/20/17
Cumulative clinical SLE manifestations, n (%)	
Mucocutaneous	43 (58.9)
Musculoskeletal	23 (31.5)
Serosal	10 (13.7)
Renal	41 (56.2)
Cardiac	13 (17.8)
Hematological	42 (57.5)
Neuropsychiatric	18 (24.7)
APS	12 (16.4)
Laboratory features	
Presence of anti-nuclear antibody, n(%)	72 (98.6)
Positive anti-DNA antibody (>20 UI/ml), n (%)	43 (58.9)
Anti-dsDNA antibodies (UI/ml), median (mean ± SD)	149 ± 323
Complement	
Low C3, n (%)	35 (47.9)
Low C4, n (%)	29 (39.7)
C3, g/l, median (mean ± SD)	0.76 ± 0.32
C4, g/l, median (mean ± SD)	0.13 ± 0.10
aPLs (any positive)	21 (28.7)
LA positive	17 (23.2)
ESR (mm/h)	19.9 ± 23.4
hs-CRP (mg/L)	6.4 ± 16.5
Creatinine (μmol/L)	81.8 ± 92.3
Treatment, n (%)	
Glucocorticoids	60 (82.2)
HCQ	59 (80.8)
Additional immunosuppressive drugs	52 (71.2)

Presence of anti-nuclear antibody (ANA) was positive in 72 (98.6%) patients. 43 (58.9%) patients had positive anti-DNA antibody with a mean value of 149 ± 323 UI/ml. Low complement C3 level was found in 35 (47.9%) patients, and low complement C4 level was found in 29(39.7%) patients. 21 (28.7%) patients had at least one type of aPL positivity, and 17 (23.2%) patients had positive LA. Laboratory characteristics were summarized in **Table 1**.

Glucocorticoids were administered to 60 (82.2%) patients at the time of evaluation. Hydroxychloroquine was used in 59 (80.8%) patients. Additional immunosuppressive drugs were given to 52 (71.2%) patients.

On ophthalmological examinations, the mean BCVA in SLE group was 0.06 ± 0.05 logMAR, while BCVA in the control group was 0.00 ± 0.24 logMAR (*p*=0.02). The mean spherical equivalent was -1.70 ± 1.78D in the SLE group, and -1.55 ± 1.45D in the control group (*p*=0.854). Among all included SLE patients, 64 (86.5%) patients had normal fundus; 7 (9.5%) patients had cotton wool spots, retinal hemorrhage, or occlusion of small vessels; 1 (1.4%) patient had optic disc edema, 2(2.7%) patients had disrupted ellipsoid zone.

### SS-OCT parameters

**Figure 2:**
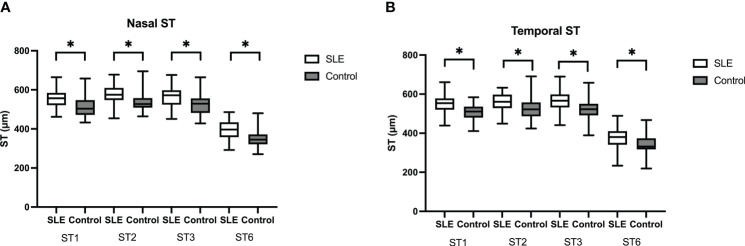
Box-and-whisker plot showing scleral thickness in SLE group and the control group. **(A)** Scleral thickness was significantly thicker at nasal ST1, ST2, ST3, and ST6 sites in SLE group than control group. **(B)** Scleral thickness was significantly thicker at temporal ST1, ST2, ST3, and ST6 sites in SLE group than control group. The boxes represent medians and interquartile ranges, with the lines in the middle of the boxes corresponding to the median values. P values were obtained by the Manne-Whitney U test. *p<0.05.

No significant differences were found in central cornea thickness, anterior chamber depth, central macular thickness and subfoveal choroidal thickness between SLE group and control group. Scleral thickness at 1mm, 2mm, 3mm, and 6mm from scleral spur nasally and temporally were compared between the two groups. Mean nasal and temporal scleral thickness were also compared between groups. Scleral thickness in the SLE group was thicker than that in the control group (**Figure 2**). The difference was significant at all measured sites (all p<0.01) (**Table 2**).

**Table 2:** Ophthalmologic characteristics and SS-OCT parameters in SLE group and control group.

	SLE patients (n=73)	Control (n=48)	P Value
BCVA (LogMAR)	0.05 ± 0.19	0.00 ± 0.24	0.019^*^
SE (Diopter)	-1.72 ± 1.79	-1.55 ± 1.45	0.800
CCT (μm)	518 ± 37	510 ± 60	0.064
ACD (mm)	3.06 ± 0.30	3.00 ± 0.20	0.385
CMT (μm)	236 ± 33	248 ± 47	0.849
SFCT (μm)	328 ± 102	311 ± 112	0.596
Nasal-ST1 (μm)	556 ± 45	513 ± 49	<0.001^*^
Nasal-ST2 (μm)	578 ± 44	535 ± 42	<0.001^*^
Nasal-ST3 (μm)	564 ± 57	522 ± 48	<0.001^*^
Nasal-ST6 (μm)	396 ± 48	350 ± 41	<0.001^*^
Mean nasal ST (μm)	525 ± 39	480 ± 35	<0.001^*^
Temporal-ST1 (μm)	548 ± 41	510 ± 42	<0.001^*^
Temporal-ST2 (μm)	558 ± 46	523 ± 50	<0.001^*^
Temporal-ST3 (μm)	565 ± 52	523 ± 50	<0.001^*^
Temporal-ST6 (μm)	374 ± 49	341 ± 44	<0.001^*^
Mean temporal ST (μm)	512 ± 38	474 ± 36	<0.001^*^

OCT, optical coherence tomography; BCVA, best corrected visual acuity; SE, spherical equivalence; CCT, central corneal thickness; ACD, anterior chamber depth; CMT, central macular thickness; SFCT, subfoveal choroidal thickness; ST, scleral thickness.

*Analysis of Mann-Whitney analysis between groups.

### Scleral thickness and disease duration

**Figure 3:**
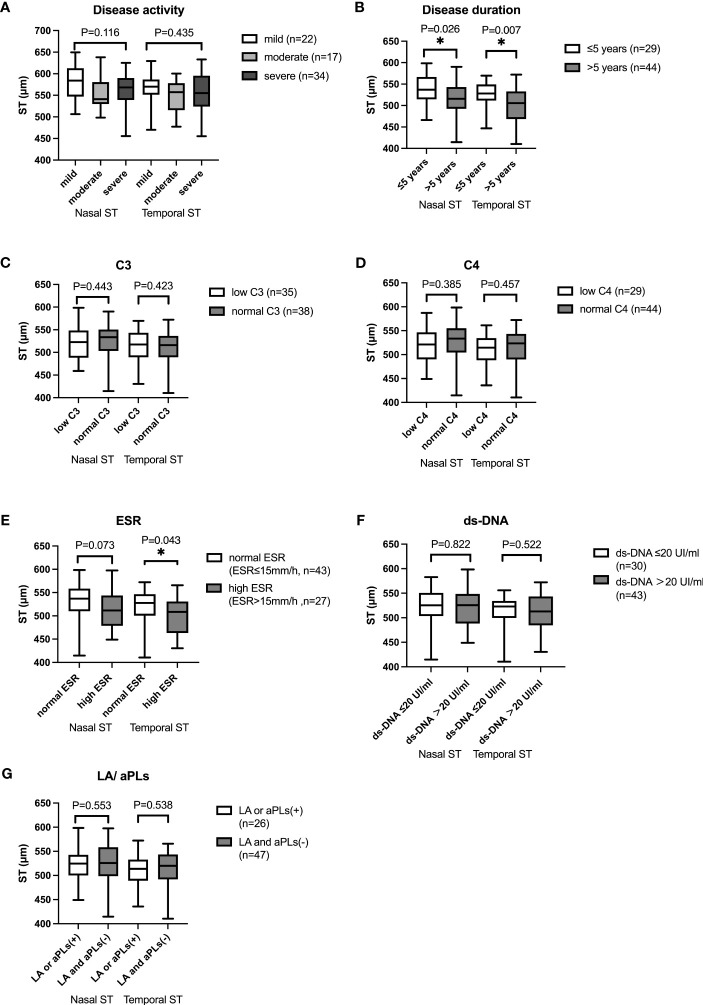
Box-and-whisker plot showing mean nasal and temporal scleral thickness in SLE patients with different subgroups. **(A)** Mean nasal and temporal scleral thickness in SLE patients with different disease activity. **(B)** Mean nasal and temporal scleral thickness in SLE patients with different disease duration. Mean nasal and temporal ST were thicker in SLE patients with disease duration ≤5 years than those in patients with disease duration of longer than 5 years (p<0.05). **(C)** Mean nasal and temporal scleral thickness in SLE patients with different C3 level. **(D)** Mean nasal and temporal scleral thickness in SLE patients with different C4 level. **(E)** Mean nasal and temporal scleral thickness in SLE patients with different ESR. Mean temporal ST was higher in SLE patients with normal ESR than that in patients with higher ESR (p<0.05). **(F)** Mean nasal and temporal scleral thickness in SLE patients with different ds-DNA. **(G)** Mean nasal and temporal scleral thickness in SLE patients with different LA/aPLs. The boxes represent medians and interquartile ranges, with the lines in the middle of the boxes corresponding to the median values. * p<0.05.

29 SLE patients had disease duration less than 5 years, 44 patients had disease duration longer than 5 years. Scleral thickness was significantly higher in patients with ≤5 years of disease duration compared with those with > 5 years of disease duration (mean temporal: 537 ± 36mm vs 514 ± 41mm, p=0.026; mean nasal: 526 ± 31mm vs 501 ± 39mm, p=0.007) (**Figure 3B**).

### Scleral thickness and laboratory markers

Mean nasal and temporal scleral thickness were compared among patients with different disease activity according to SLEDAI-2K. No significant difference was found in mean nasal or temporal scleral thickness with different SLE activity (Mean nasal: mild 562 ± 42μm, moderate 581 ± 41μm, severe 556 ± 39μm, p=0.116; Mean temporal: mild 556 ± 42μm, moderate 566 ± 39μm, severe 548 ± 40μm, p=0.435). We then compared mean nasal and temporal scleral thickness in SLE patients with different serum level of C3, C4, ESR, and ds-DNA. Temporal scleral thickness was higher in patients with normal ESR than in patients with high ESR (Mean temporal Normal ESR: ESR<15mm/h 515 ± 40μm, High ESR: ESR≥15mm/h 500 ± 39μm, p=0.043). The result showed that mean nasal and temporal scleral thickness was not significantly different in patients with different levels of C3, C4, and ds-DNA. No significant difference was found in patients with positive LA or aPLs and patients with negative LA and aPLs (**Figure 3**).

### Scleral thickness and systemic involvement

**Figure 4:**
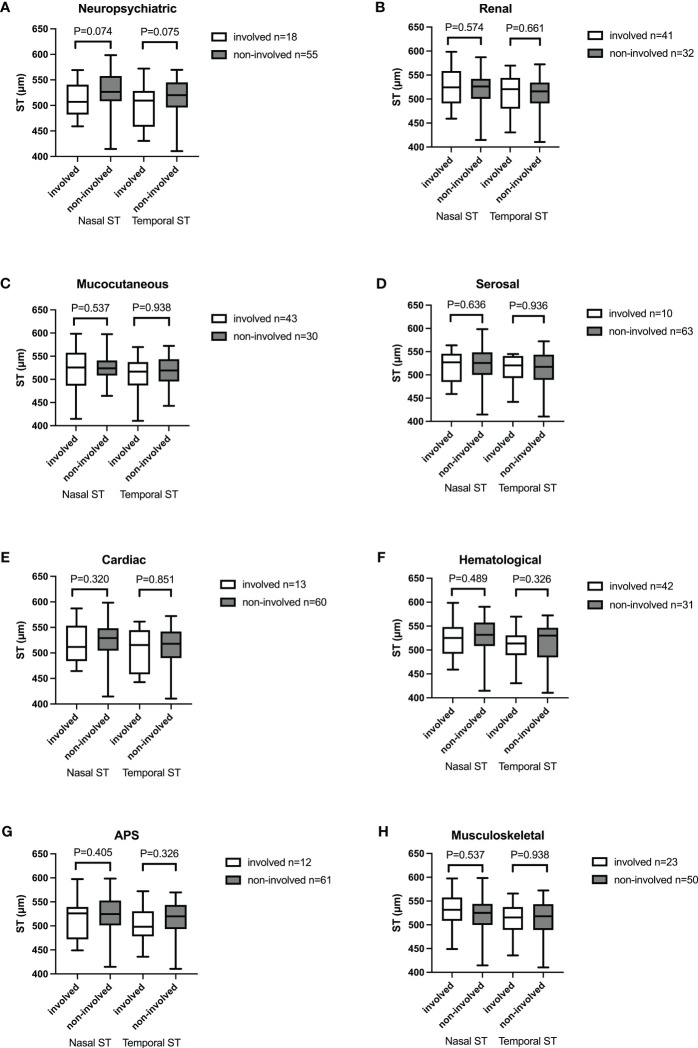
Box-and-whisker plot showing mean nasal and temporal scleral thickness in SLE patients with different organ involvement. **(A)** Neuropsychiatric. **(B)** Renal. **(C)** Mucocutaneous. **(D)** Serosal. **(E)** Cardiac. **(F)** Hematological. **(G)** APS. **(H)** Musculoskeletal. No significant difference was found between groups. The boxes represent medians and interquartile ranges, with the lines in the middle of the boxes corresponding to the median values.

Scleral thickness in patients with or without organ or systemic involvement was compared. Mean nasal and temporal scleral thickness were thinner in patients with neuropsychiatric involvement than those without neuropsychiatric involvement, but the differences were not statistically significant (Mean nasal: with neuropsychiatric involvement 511 ± 35, without neuropsychiatric involvement 529 ± 39; p=0.074; Mean temporal: with neuropsychiatric involvement 489 ± 40, without neuropsychiatric involvement 516 ± 36, p=0.075). We also compared scleral thickness between groups in patients with or without renal, mucocutaneous, serosal, cardiac, hematological involvement, and secondary APS and found no significant difference between groups (**Figure 4**).

## Discussion

SLE is a chronic autoimmune disease capable of causing damage to multiple organs and systems, including the eye. Eye involvement can be diagnosed in approximately one-third of SLE patients (24). Any part of the visual system can be affected, including the conjunctiva, cornea, sclera, retina, uvea tract, optic nerve, and the orbit. Scleritis or episcleritis is relatively uncommon in SLE with a prevalence of 2.4% (25), and is usually correlated with the activity of disease. Some studies have reported scleritis and episcleritis in SLE (24, 26), but studies evaluating scleral thickness in SLE patients are limited (18). In the current study, scleral thickness was evaluated using AS OCT in eyes with SLE in comparison with normal control eyes. SLE eyes showed significantly thicker scleral at multiple sites than the control eyes. Scleral thickness was thicker in SLE eyes with disease duration of less than 5 years compared with SLE eyes with disease duration of more than 5 years. No significant difference of scleral thickness was found between groups with different levels of laboratory markers and organ involvement. To the best of our knowledge, this study provides the first report with comprehensive comparison of scleral thickness in SLE patients with different laboratory parameters and organ involvement.

AS OCT has been applied to evaluate scleral changes and scleral thickness in some recent researches. Watson et al. found collagen fiber separation in nonnecrotizing nodular scleritis and tissue necrotizing in necrotizing scleritis with the help of AS OCT and histopathological studies (27). Buckhurst and Read et al. measured scleral thickness at different distances from the scleral spur in children and normal populations (28, 29). Imanaga and colleagues measured scleral thickness with AS OCT in central serous chorioretinopathy (CSC) and hypothesized that thick sclera may have a role in the pathogenesis of CSC (21). Later studies have also shown that AS OCT is a useful tool to detect changes of sclera in various diseases (22), (30). A recent study using spectral-domain OCT with an AS module by Kaya et al. found that scleral thickness was thicker in SLE patients compared with normal control eyes. (18) In Kaya’s study, temporal scleral thickness at distances of 1, 2, 3, 4, and 5mm from scleral spur were measured and the ST ranges from 587.46 ± 49.22μm to 627.14 ± 48.59μm in SLE patients. In our study, scleral thickness was thicker than control eyes at all measured locations, which was in concordance with Kaya’s study. Moreover, we measured both temporal and nasal ST with extended site of 6mm from scleral spur. Our result provided a more comprehensive description of the scleral thickness both in SLE patients and normal control eyes. Besides, we used the anterior module of the SS-OCT for sclera imaging and calibration. The SS-OCT has better tissue penetration and axial resolution than SD-OCT. The boarder of sclera might be difficult to recognize in SD-OCT images, but with the help of SS-OCT, the border of sclera can be distinguished from the conjunctiva and connective tissues easily.

The scleral is rich in collagen fibers and elastic fibers (10). The episcleral complex is a well vascularized connective tissue that blends imperceptibly with the scleral stroma. Scleral stroma is supplied by episcleral and choroidal vascular networks rather than vessels passing through it. Episcleritis affects superficial episcleral vasculature, while scleritis affects deep episcleral vessels. Previous researches showed thickened scleral on AS OCT in episcleritis and scleritis (11, 13, 31), and the thickening was mostly caused by a thickened episcleral layer (11, 31). Hypo-reflective spaces were found in Inflamed scleral tissue with AS OCT and UBM, and the hypo-reflective areas were believed to represent edematous thickening of tissues with infiltration of inflammatory cells (32, 33) or the unravelling of collagen fibris, which has been shown with histopathological studies (11, 12, 34). Accumulation of immune complexes has also been reported and the complement systems were likely to be involved (35, 36). In our study, we enrolled SLE patients without episcleritis or scleritis. Despite thickened scleral, no hypo-reflective areas were found in our patients. We think the thickened sclera in SLE patients might be subclinical changes caused by collagen fiber swelling or deposition of immune complexes.

A previous study showed disease activity and duration were not correlated with scleral thickness in SLE patients (18). The result of our study agreed with previous research that there was no difference in sclera thickness in patients with different SLEDAI-2k score, but disease duration has influenced sclera thickness in SLE patients. In our study, scleral thickness in patients with disease duration of less than 5 years was thicker than that in patients with disease duration of longer. Some previous studies have shown that scleral changes with aging, glaucoma, and myopia. Extracellular matrix (ECM) remodeling including reformation of collagen structure and regulation of scleral composition may be one of the key contributors of disease progression (37–40). One hypothesis for the difference in scleral thickness in patients with different disease duration was that subclinical changes of sclera happened slowly over a long period of time. Extracellular matrix, particularly collagen remodeling may be active in the early few years of disease. As the disease prolonged, ECM remodeling subdued and resulted in a relatively thin sclera. Another hypothesis is that the initial inflammatory status of SLE resulted in a thickened sclera at the early stage of SLE. However, histopathological studies are needed to further elucidate the pathogenesis of sclera thickness changes in SLE patients.

ESR is an inflammatory marker and has been known to be regularly elevated in active SLE (41). We have described a thinner mean nasal and temporal scleral thickness in SLE patients with ESR>15mm/h than those with ESR ≤15 mm/h. A previous study by Yazici et al. found biomechanical alterations of cornea in SLE patients and the authors attributed the changes to corneal collagen lysis and thinning caused by local antigen-antibody reaction (42). Similarly, collagen lysis and thinning in inflammatory status may cause a thinning scleral tissue, as is shown in our study. However, the relationship between ESR and scleral changes were not clear. More studies are needed to provide more evidence about scleral changes in SLE patients.

In the present study, we also compared scleral thickness in SLE patients with or without different organ or systemic involvement. No significant difference was found between patients with or without neuropsychiatric, renal, mucocutaneous, serosal, cardiac, hematological, APS, or musculoskeletal involvement. The result may indicate that changes of sclera is independent of other organ involvement.

Of noted, most of the SLE patients enrolled in our study were under the treatment of cortical steroid, or combined with additional immunosuppressive drugs. Previous study shown that steroid may induce greater senescence in scleral fibroblast cells compared with controls. The fibroblast cells proliferated slower, grew larger, and migrated less when treated with steroid (43) However, the mechanism of steroid induced scleral changes was not clear and further studies are needed to elucidate the underlying mechanism. As for the influence of other immunosuppressive drugs to the scleral tissue, there is a lack of evidence showing how immunosuppressive drugs may cause scleral changes. However, this is a question worth studying since immunosuppressive drugs may cause changes of microcirculation or cellular components of the scleral tissue and eventually result in structural changes of the sclera. More studies are needed to provide us with evidence of how immunosuppressive drugs may influence the sclera structure.

There are several limitations of the current study. The study was a cross-sectional study including retrospective data collection. Studies with follow-ups would provide more information about the dynamic changes of sclera in SLE patients, which was not done in the present study. Although the sample size is small, the study provided structural data of sclera obtained with SS-OCT in the largest series of SLE that has been published. Besides, the study population included SLE patients with different disease severity from both outpatient clinics or inpatient ophthalmic consultations.

## Conclusion

In summary, our study showed a thicker sclera in SLE patients without clinically active scleritis and episcleritis than normal controls. Scleral thickness was thicker in patients with ≤5 years of disease duration compared with those with > 5 years of disease duration. Temporal scleral thickness was thinner in patients with higher ESR. The changes of scleral thickness might be related to subclinical inflammation and extracellular matrix remodeling. SS OCT can detect asymptomatic structural changes in SLE patients and may be a useful tool in the evaluation of early scleral abnormality.

## Data availability statement

The raw data supporting the conclusions of this article will be made available by the authors, without undue reservation.

## Ethics statement

The studies involving humans were approved by the institutional review board of PUMCH. The studies were conducted in accordance with the local legislation and institutional requirements. The participants provided their written informed consent to participate in this study.

## Author contributions

LC: Conceptualization, Writing – original draft. LM: Data curation, Writing – review & editing. LS: Investigation, Writing – review & editing. YC: Writing – review & editing.
